# Preparation of Ion^2+^-COS/SA Multifunctional Gel Films for Skin Wound Healing by an In Situ Spray Method

**DOI:** 10.3390/md20060401

**Published:** 2022-06-18

**Authors:** Liqi Chen, Tingting Guo, Chao Shi, Kun Zhang, Shenghao Cui, Di Zhao, Faming Yang, Jingdi Chen

**Affiliations:** 1 Marine College, Shandong University, Weihai 264209, China; clychee0307@163.com (L.C.); gtt1314xf@163.com (T.G.); shichao19834031211@163.com (C.S.); sdtzzk2021@163.com (K.Z.); csh1473@163.com (S.C.); mjqyhhdzdys@163.com (D.Z.); 2 Shandong Laboratory of Advanced Materials and Green Manufacturing, Yantai 265599, China

**Keywords:** marine polysaccharide, metal ion-COS chelate, in-situ spray, antibacterial activity, skin wound healing

## Abstract

The rapid preparation of safe and efficient wound dressings that meet the needs of the entire repair process remains a major challenge for effective therapeutic wound healing. Natural, sprayable Ion^2+^-COS/SA multifunctional dual-network gel films created by the in situ coordination of chitooligosaccharide (COS), metal ions and sodium alginate (SA) using casting and an in-situ spray method were synthesized. The gel films exhibited excellent physicochemical properties such as swelling, porosity and plasticity at a COS mass fraction of 3%. Furthermore, at this mass fraction, the addition of bimetallic ions led to the display of multifunctional properties, including significant antioxidant, antibacterial and cytocompatibility properties. In addition, experiments in a total skin defect model showed that this multifunctional gel film accelerates wound healing and promotes skin regeneration. These results suggest that the sprayable Ion^2+^-COS/SA multifunctional pro-healing gel film may be a promising candidate for the clinical treatment of allodermic wounds.

## 1. Introduction

As the largest human organ, the skin not only serves as a natural barrier to the body from external damage and microbial invasion but also prevents the loss of nutrients from the body [1]. At this stage, the problem of skin damage caused by trauma, surgery and chronic diseases needs to be addressed urgently [2]. Failure to heal promptly and effectively usually leads to chronic wounds, and may also cause problems including poor circulation, a lack of oxygen, bacterial infections, and high pH levels of white blood cells [3,4,5]. Inspired by the concept of moist wound healing, gel film dressings provide a moist healing environment that mimics the extracellular matrix and serves to allow gas exchange [6]. Not only that, properly designed gel film structures and functions can activate wound biological activities such as cell proliferation, infiltration and vascularization to promote tissue reconstruction and wound healing, and are considered ideal for wound healing [7,8].

Among many gel films, the existence of double-network gel films can ensure sufficient mechanical properties of gel films, but the traditional preparation process often adds a variety of chemical cross-linking agents. The addition of chemical cross-linking agents not only leads to the existence of certain toxic side effects of gel films but also tends to destroy their biocompatibility and biodegradability [9,10]. Thus, the development of fully physically cross-linked double-network gel films is particularly important, not only to avoid the excessive use of toxic chemicals, but also to promote the wide application of gel films in biomedical and other fields.

SA is a natural linear anionic polysaccharide from brown algae that can exhibit simple gelation through interaction with divalent cations. COS has good bioadhesive, antimicrobial and hemostatic properties as a wound dressing [9,11]. The use of both as gel film precursors allows the preparation of natural and safe double-network gel films, which are widely used in the biomedical field [12]. Furthermore, by addition of additional inorganic components with specific bioactivity, such as calcium, zinc and strontium, they can be combined highly synergistically with alginate to form physical double-network gel films, thus improving their physicochemical properties and bioactivity [13].

Herein, we successfully prepared a Ion^2+^-COS/SA physically cross-linked double-net gel film (referred to as COS/SA/M^2+^ PDF) with various functions, including promoting wound healing by casting and the in-situ spray method. This preparation method overcomes the problems of the traditional preparation process and the long preparation time. More interestingly, although it is made by the in situ spray method, the addition of several different divalent metal ions overcomes the poor mechanical properties and enables it to exhibit excellent antibacterial properties and cellular activity [14]. The content of metal ions was controlled within the safe range; thus, the safety of the gel films could be guaranteed. Meanwhile, in vivo healing promotion experiments have demonstrated that this metal ion cross-linked gel film can significantly improve the wound healing process. Therefore, this gel film has great potential to be used in the promotion of skin wound healing due to its rapid and convenient preparation method and the natural safety of the film itself.

## 2. Results and Discussions

### 2.1. Preparation Mechanism of COS/SA/M^2+^ PDF

In order to overcome the problems of its low degradability and low biocompatibility, and to improve patient compliance, we designed in situ spray gel films to develop effective drug delivery devices. It was shown that the large number of amino and hydroxyl groups in COS are good binding sites for metal ions [15]. The cage-like molecular structure formed by intermolecular ammonia and salt bonds can enhance the film’s application in the production of biopharmaceuticals and healthcare products, in food processing, and in other fields [16].

Thus, the COS/SA/M^2+^ PDF consisted of a COS/SA network and an SA/Ion^2+^ network. These two physical cross-linked networks were formed in the following order: the first network was formed by the electrostatic interaction between the protonated amino group of COS and the carboxyl group of SA; the second network was the cross-linking of SA and Ion^2+^. The synergistic effect of the two networks will maintain the integrity of the network structure and prolong the service life of the gel films [17].

### 2.2. Chemical Structure and Property Analysis of COS/SA/M^2+^ PDF

#### 2.2.1. Surface Appearance

**Figure 1:**
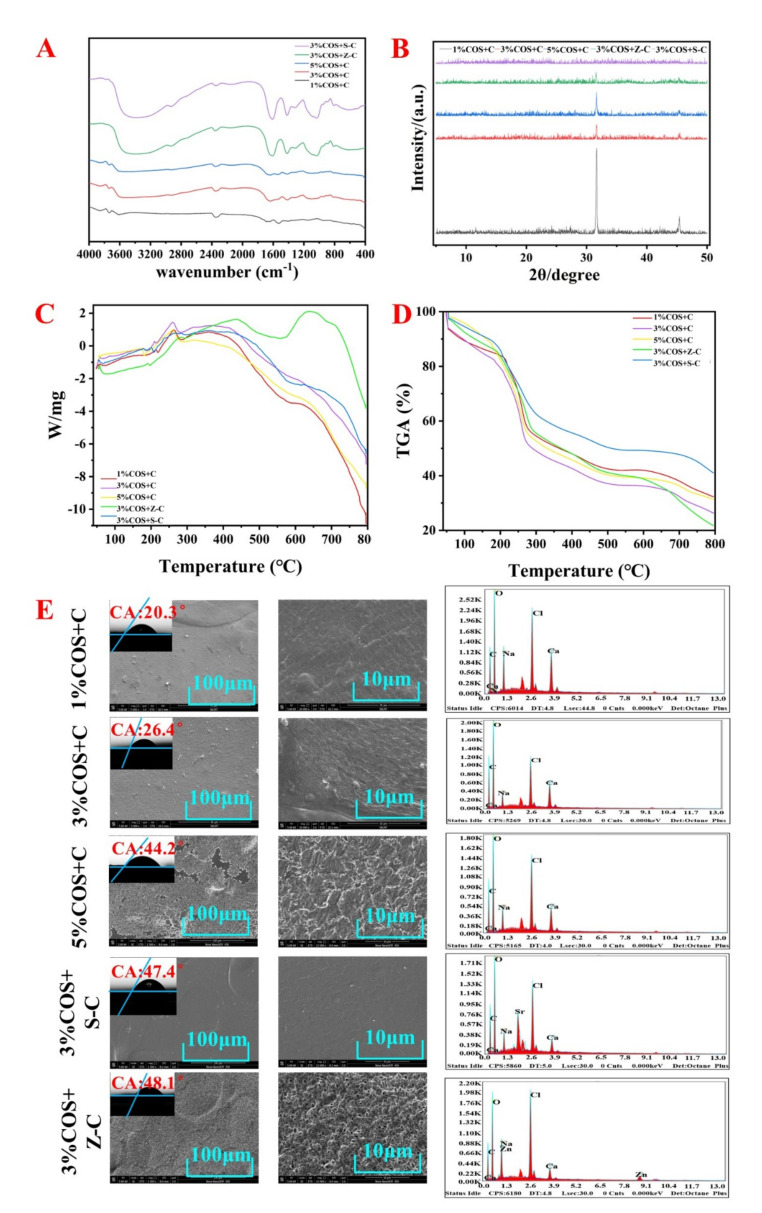
Characterization of Ion^2+^−COS/SA gel films. (**A**) FTIR spectra of Ion^2+^−COS/SA gel films. (**B**) XRD spectrum of Ion^2+^−COS/SA gel films. (**C**,**D**) TGA patterns of Ion^2+^−COS/SA gel films. (**E**) SEM morphology and energy spectrum analysis of Ion^2+^−COS/SA gel films.

The surface morphologies of these gel films were determined by SEM (Figure 1E). It can be clearly observed in the figure that the 3% COS + Z-C and 3% COS + S-C group both formed a double network of which the cross-section showed more and smaller lattices with a denser structure. Numerous studies have shown that the porosity and connectivity of gel film structures are important for biological applications [18].

#### 2.2.2. FTIR

FTIR spectroscopy was used for the study of the intermolecular interactions between SA, COS, and metal ions [8]. In Figure 1A, the peaks at 3404 cm^−1^ were attributed to the stretching vibrations of O-H and N-H bonds, the absorption peak at 1070 cm^−1^ was from the bending vibrations of C=O, and the absorption peaks near 1645 cm^−1^ and 1625 cm^−1^ were attributed to amide band I and amide band II. The absorption peaks of the 3% COS + Z-C and 3% COS + S-C groups were enhanced at 1674 cm^−1^ and 1623 cm^−1^ compared to the other groups. The 1% COS + C, 3% COS + C, and 5% COS + C groups were enhanced with increasing COS concentrations. This was attributed to the stretching vibration of double-bond to single-bond -COO and C=O in the amide group, and the bending vibration of N-H in the amide group [19]. The absorption peaks at 1612 cm^−1^ and 1415 cm^−1^ shift to shorter wavelengths with increasing COS concentrations in 1% COS + C, 3% COS + C, and 5% COS + C, indicating that single-bonded -COO^−^ and single-bonded -NH_3_^+^ interact with Ca^2+^ [20]. The above situation indicates that the metal ions are successfully attached to SA. The shift of the amide band in metal ion cross-linked gel films, indicating the presence of intermolecular hydrogen bonds, is consistent with the large amount of free energy detected in XRD [21,22]. In conclusion, the FTIR results indicate the successful preparation of these metal ion cross-linked gel films.

#### 2.2.3. XRD

In the XRD plot, there was a comprehensive main peak at 30° which was clearly generated by the crystalline phase (Figure 1B). As the COS content increased, the sample changed from the crystalline phase to the amorphous form, and the diffraction peak obtained gradually decreased, which was consistent with the results of the DSC curve. The diffraction peaks of both the Sr^2+^-containing and Zn^2+^-containing groups were lower. The pleasant surprise was that the transformation of the Zn^2+^-containing group was complete, and the resulting powder was amorphous. Therefore, the cross-linking of polymers suppresses the adjacent stacking of chains by reducing the degree of freedom of the 3D conformation, and by inhibiting or even avoiding the development of crystalline regions [23]. From the FTIR results it can be seen that there was a large amount of hydrogen bond formation, which also further leads to the presence of free energy. Generally speaking, SA and COS in the gel film network usually show a semi-crystalline structure [24].

#### 2.2.4. Contact Angle

The contact angle measurements of each film were less than 50° (Figure 1E), reflecting that each COS/SA/M^2+^ PDF had a more excellent hydrophilicity, which contributes to wound recovery according to the wet wound healing theory [25].

#### 2.2.5. Inflationary Behavior

**Figure 2:**
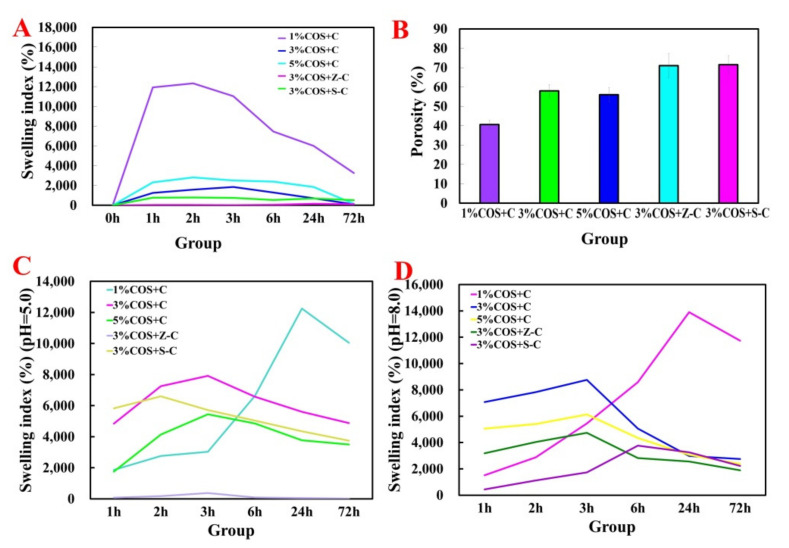
Swelling behavior and porosity of Ion^2+^−COS/SA gel films at different pH. (**A**) Expansion behavior under neutral conditions of Ion^2+^−COS/SA gel films. (**B**) Porosity of Ion^2+^−COS/SA gel films. (**C**) Swelling behavior under pH 5.0 conditions of Ion^2+^−COS/SA gel films. (**D**) Swelling behavior under pH 8.0 conditions of Ion^2+^−COS/SA gel films.

It was observed that the gel films of all of the groups swelled regularly until they reached an equilibrium value (Figure 2A). The equilibrium swelling rate of the 1% COS + C group was significantly higher than that of the other groups, exhibiting a rapid water uptake (up to 12,000% at 2 h). Compared with the single-ion group, the introduction of Sr^2+^ and Zn^2+^ enhanced the stability of the gel films, which led to an increase in the cross-link density of the gel film fibers, resulting in a decrease in the swelling multiplier [26]. This may also be due to the fact that the SA in the multi-ionic gel films can partially dissolve in water and precipitate out during the swelling process, resulting in a decrease in the added mass of the gel films, which eventually leads to a decrease in swelling.

In order to further verify its usefulness in promoting wound healing in practical applications, its swelling behavior was evaluated at pH 5.0 versus pH 8.0 (Figure 2C,D). It was found that the 3% COS + Z-C group still maintained a good stability, and the trend of the other groups also remained consistent with the neutral conditions, which is favorable for practical promotion.

#### 2.2.6. Porosity

A proper pore structure facilitates nutrient transport and cell adhesion, as well as water vapor penetration [27]. The gel film corresponding to the polyionic group had the largest porosity (Figure 2B), which could ensure its adequate permeability as a wound dressing. This result is in high agreement with the findings of the SEM morphological analysis.

#### 2.2.7. Thermal Stability

In order to estimate the thermal stability of the gel films, thermogravimetric analysis was performed. The three gel films exhibited essentially similar decomposition trends, which could be divided into three stages. Figure 1C,D showed that the range of 35–250 °C was accompanied by about 30–50% weight loss, probably due to the electrostatic interaction between the amino group on COS and the carboxyl group on SA by warming, and also due to the loss of chelation between metal ions and SA, as well as the decomposition of COS. Interestingly, up to the decay stage at 800 °C, the Sr^2+^-containing group showed about 80% weight loss, while the Zn^2+^-containing group showed about 55% loss, which was caused by the evaporation of some volatiles from thermal decomposition and the burning of the polymer chains, and the lesser loss of the Zn^2+^-containing group proved its greater stability to meet the needs of medical materials [28].

### 2.3. Mechanical and Rheological Analysis of COS/SA/M^2+^ PDF

**Figure 3:**
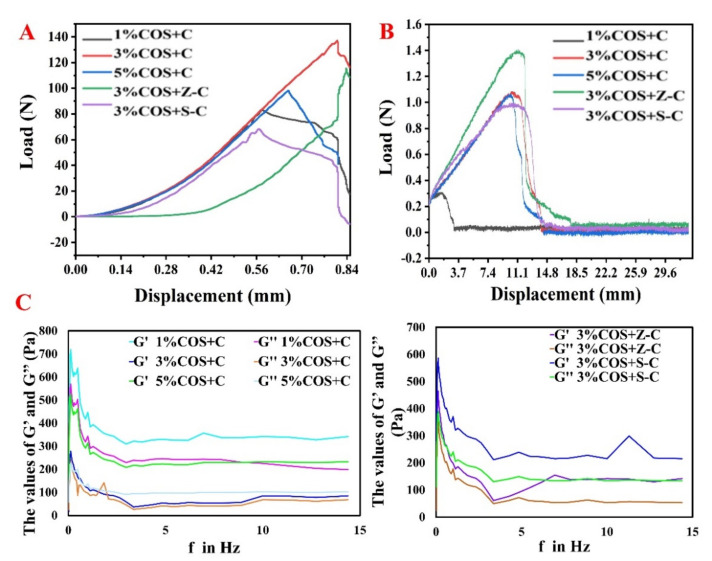
Results of the mechanical and rheological analysis of Ion^2+^−COS/SA gel films. (**A**) Tensile strength and deformation curves of Ion^2+^−COS/SA gel films. (**B**) Fracture strength of Ion^2+^−COS/SA gel films. (**C**) Dynamic rheological behavior of Ion^2+^−COS/SA gel films.

The gel films must have superior mechanical properties because the trauma surface conforms to human movement in practical applications. As shown in Figure 3A,B, the tensile strengths were the 3% COS + Z-C, 1% COS + C, 5% COS + C, 3% COS + C, and 3% COS + Z-C groups, from the largest to the smallest. The maximum tensile stress of the gel film of the 3% COS group was 140 N; this group had good tensile strength and elasticity. The Zn^2+^-containing group could withstand a greater tensile force, proving that the addition of Zn^2+^ increased the cross-linking and enhanced the mechanical properties of the gel film, indicating that it could be applied to the skin surface to accommodate human movement without causing injury. It is worth noting that it could also be used to develop wound dressings that meet different mechanical property requirements.

The rheological properties of the hydrogel films were analyzed as shown in Figure 3C. The storage modulus (G’) can be used to reflect the elasticity of the material, and the loss modulus (G’’) can be used to characterize its viscosity. Figure 3C shows that the storage modulus of the film was basically higher than the loss modulus, indicating the better elastic properties of the gel films. This is also consistent with the mechanical property test results, and reflects the rheological properties of the general gel film as well.

### 2.4. Antioxidant Activity of COS/SA/M^2+^ PDF

**Figure 4:**
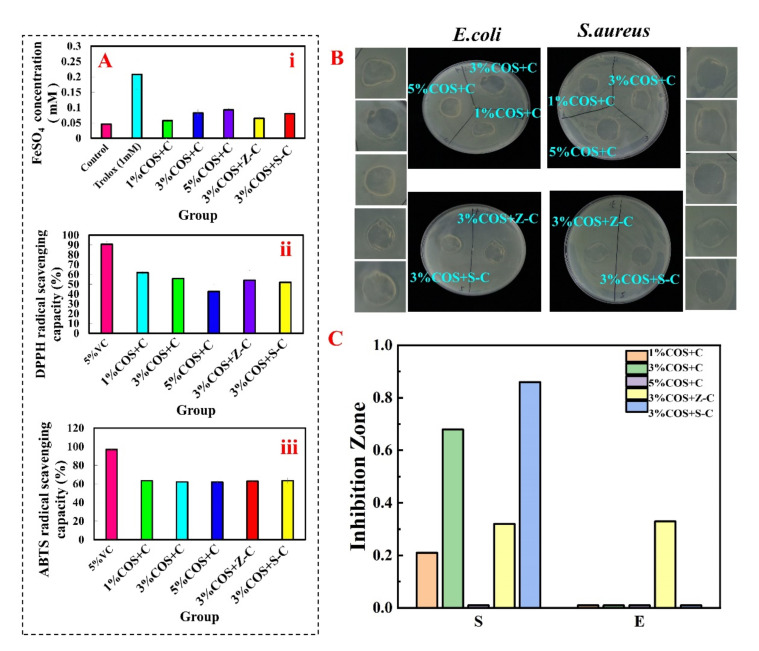
In vitro activity evaluation of Ion^2+^−COS/SA gel films. (**A**) In vitro antioxidant performance evaluation of Ion^2+^−COS/SA gel membranes, from top to bottom: Fe^2+^ chelating activity, DPPH radical scavenging activity, and ABTS radical scavenging activity. (**B**) Inhibition circles formed by the action of different Ion^2+^-COS/SA gel films. The left column, from top to bottom, shows 1% COS + C, 3% COS + C, 5% COS + C, 3% COS + Z-C, and 3% COS + S-C, corresponding to the inhibition circles (*E. coli*); the right column, from top to bottom, shows 1% COS + C, 3% COS + C, 5% COS + C, 3% COS + Z-C, and 3% COS + S-C, corresponding to the inhibition circles (*S. aureus*). (**C**) Histogram of the inhibition circles of different Ion^2+^-COS/SA gel films.

Fe^2+^ chelating ability, ABTS radical scavenging activity and DPPH radical scavenging activity are widely used to evaluate the antioxidant properties of functional substances [29]. The in vitro antioxidant activity results of the gel films of each group are shown in Figure 4A. Notably, each Ion^2+^-COS/SA multifunctional pro-healing gel film had good scavenging and reducing ability for all three, but all of them were lower than the positive control group. This proves that the metal ion complex gel films have good antioxidant ability, which provides an idea for the preparation of versatile natural gel films.

### 2.5. Antibacterial Properties of COS/SA/M^2+^ PDF

The ideal wound dressing should have good antimicrobial properties in order to reduce the number of pathogens in the wound, treat inflammation, and promote wound healing [30]. Numbers 1–5 in Figure 4B respectively correspond to the 1% COS + C, 3% COS + C, 5% COS + C, 3% COS + Z-C, and 3% COS + S-C groups. After 2 h of contact with the gel film’s surface, the inhibition circles were more pronounced against *S. aureus* in all of the groups except for the 5% COS + C group, for which there was no significant inhibition circle. Compared with the control group, the bacteria on the agar plates were largely killed, at 0.68 cm for the 3% COS + C group and 0.86 cm for the 3% COS + S-C group. For *E. coli*, the 3% COS + Z-C group had a significant inhibition circle (2.33 cm in diameter). The results showed that gel films containing different metal ions had excellent antibacterial activity against *E. coli* and *S. aureus*. In addition, the killing rate of the gel films against *E. coli* and *S. aureus* increased with the increase of the COS concentration in the 1% compared to the 3% group [14]. Compared with the 5% COS group, the antibacterial effect of the gel films gradually decreased with an increasing COS concentration (Figure 4C), which was mainly due to the decreasing gel film solubility. This result corresponds to the antioxidant function, proving that the gel films have good antibacterial activity and great potential for the effective prevention and treatment of bacterial infections in wounds.

### 2.6. Cytocompatibility of COS/SA/M^2+^ PDF

Cell viability and cell proliferation are important for wound contraction and later healing stages [31]. Wound dressings applied to the skin surface should be compatible with skin cells such as fibroblasts and keratin-forming cells [32]. In the study, keratin-forming cells (HaCaT) were selected for cytocompatibility evaluation.

**Figure 5:**
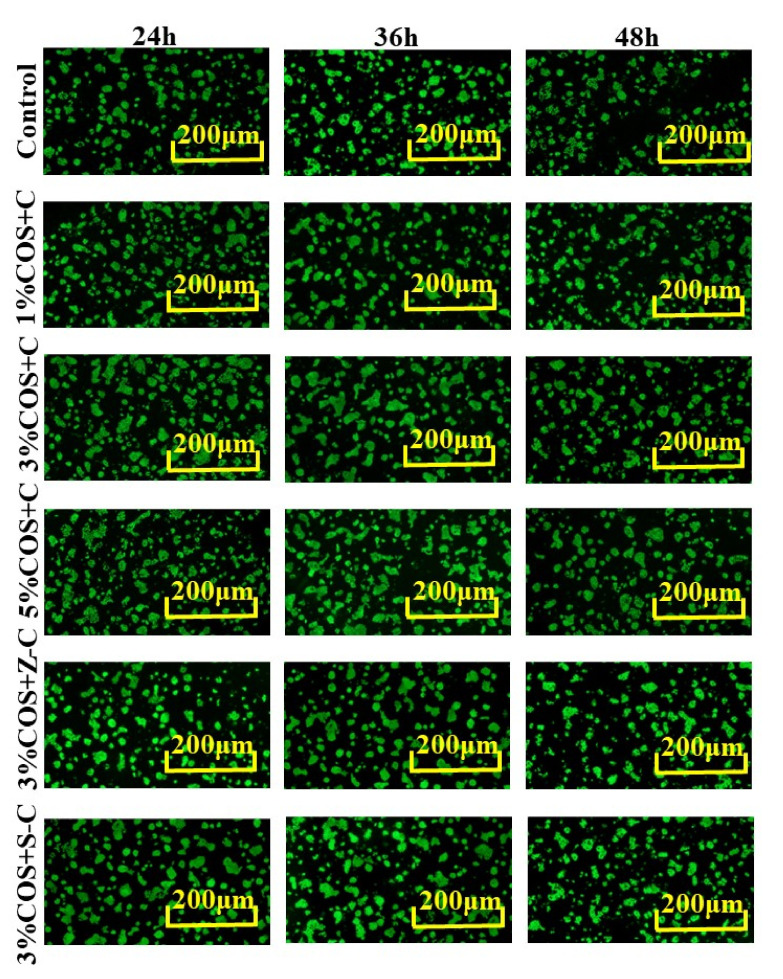
Cytotoxicity test of Ion^2+^−COS/SA gel membranes in vitro. AO/EB fluorescence staining for 24 h, 36 h and 48 h (magnification: ×5). Acridine orange (AO) fluoresces green after it’s crossing the intact cell membrane and embedding in nuclear DNA. Ethidium bromide (EB) penetrates damaged membranes, interacts with DNA, and emits orange-red fluorescence.

Figure 5 shows fluorescence micrographs of HaCaT cell viability after the co-incubation of HaCaT cells with each gel film extract for 24 h, 36 h and 48 h. Compared with the control group, the green color in the HaCaT cells cultured in each Ion^2+^−COS/SA multifunctional pro-healing gel film extract covered almost the whole image without showing any red-orange color, implying that HaCaT cells grew well in each group. The cell viability was stronger in the multi-ion group, which corresponded to the SEM structure, proving that the Ion^2+^−COS/SA multifunctional pro-healing gel film has good biocompatibility, and that it has the potential to be used as a promising biomedical material [33].

### 2.7. In Vivo Healing Promotion Experiment of COS/SA/M^2+^ PDF

The porosity of the dressing essentially affects wound healing because it determines the oxygen penetration and nutrient diffusion directly related to the wound microenvironment and cell proliferation [34]. Before conducting animal experiments, a comprehensive evaluation of all aspects of the performance of COS/SA/M^2+^ PDF was carried out, and it was found that the 3% COS + Z-C group showed good characterization in various aspects such as porosity and mechanical properties; thus, this group of gel films was selected as a dressing material in a mouse dorsal skin wound model in order to continue the evaluation of its in vivo pro-healing ability.

#### 2.7.1. Wound Healing Assessment

**Figure 6:**
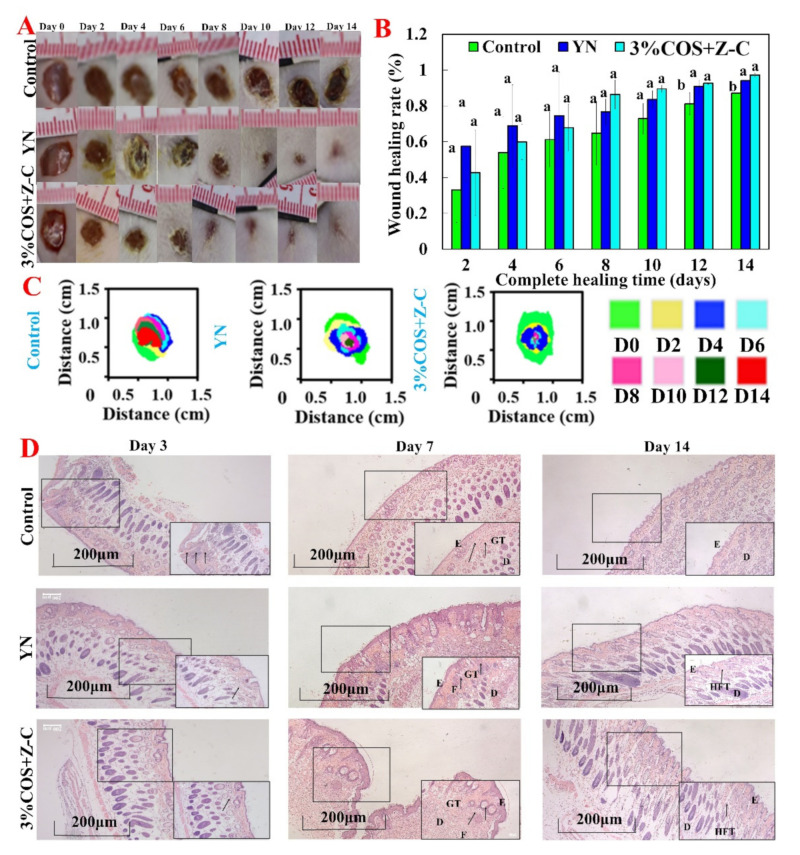
Evaluation of the wound healing-promoting ability of the Ion^2+^−COS/SA gel film. The Ion^2+^−COS/SA gel film is shown in A–C for the evaluation of wound defects in mice. (**A**) Photographs of representative wounds of mice on days 0, 2, 4, 6, 8, 10, 12 and 14. (**B**) Wound healing rate of each group (calculated every two days). The values are expressed as the mean ± standard deviation, *n* = 3. Note: The same superscript letters indicate non-significant differences (*p* > 0.05); different superscript letters indicate significant differences (*p* < 0.05). (**C**) Simulated changes in wound size and morphology at eight time points. (**D**) Representative images of H&E staining (5×). Note: Thick black arrows indicate inflammatory cell infiltration. Letters D, E, F, GT and HFT, respectively, represent dermis, epidermis, fibroblasts, granulation tissue and hair follicle tissue.

The quantitative statistical analysis of the wound healing area was performed at different time points. It is worth noting that after 4 days of treatment, the 3% COS + Z-C group showed a decrease in inflammatory cells compared to the control group, indicating an accelerated wound healing process, a decrease in the inflammatory process, and a decrease in the number of inflammatory cells. This was closely related to the significant antibacterial ability and anti-inflammatory properties of Zn^2+^ [35]. On day 8, granulation tissue formation was observed on the wounds of the 3% COS + Z-C group, and the percentage of wound closure (86.40%) was significantly reduced. Healing was essentially complete by day 14, which was consistent with the results in Figure 6B. Notably, on days 12 and 14, both the positive control and 3% COS + Z-C groups formed significant differences from the blank group (*p* < 0.05). The results indicated that the use of the 3% COS + Z-C group gel films with positively charged metal ions, a porous structure and high-water content as a wound dressing can better promote fibroblast adhesion and proliferation [36]. Numerous studies have shown that the films absorb wound exudate and provides a moist environment to promote wound healing [37].

#### 2.7.2. Histological Evaluation of the Regenerated Tissue

Wound healing is a complex process that includes the infiltration of inflammatory cells, capillary formation, the adhesion and proliferation of fibroblasts, and the deposition of collagen [38]. Histological analysis was used to assess the quality of the regenerated skin at the wound defect (Figure 6D). On day 7, inflammatory cells were significantly reduced in the 3% COS + Z-C group compared to the blank group. This is mainly because COS can inhibit the expression of inflammatory mediators and the activation of NF-κB, thereby treating the inflammatory phase and accelerating wound healing [39]. Compared with the blank group and the positive control group, the 3% COS + Z-C group showed more neovascularization, more complete dermal tissue formation, and some degree of re-epithelialization. As is consistent with the results of the wound healing experiments, it showed that metal ion-complexed gel film can effectively promote skin regeneration, and that it is a promising wound dressing.

#### 2.7.3. Expression of FGF and CD31 during Wound Healing

**Figure 7:**
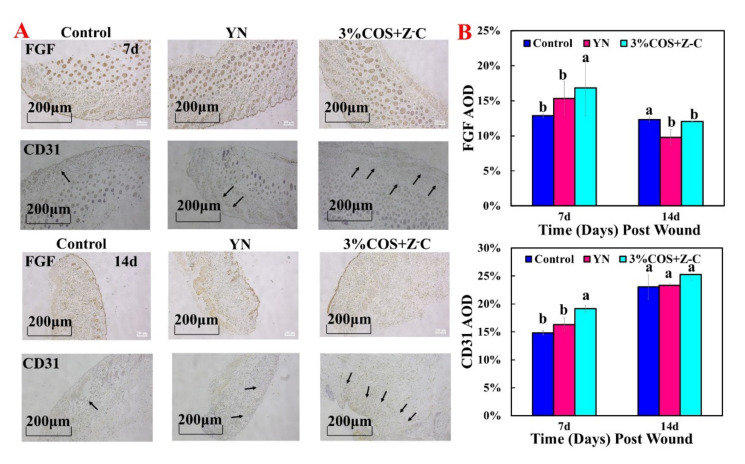
Expression of the FGF and CD31 of Ion^2+^−COS/SA gel films. (**A**) From top to bottom, representative images of the FGF and CD31 analysis on day 7; representative images of the FGF and CD31 analysis on day 14. (**B**) Quantitative immunohistochemical analysis on day 7 and day 14. Note: The same superscript letters indicate non-significant differences (*p* > 0.05); different superscript letters indicate significant differences (*p* < 0.05).

In order to analyze the effect of these metal ion gel films on cell proliferation and apoptosis, FGF and CD31 were used to detect the related protein content, in order to speculate as to its promoting effect (Figure 7A). The levels were higher on day 7, with a significant difference between the 3% COS + Z-C group and the other two groups (*p* < 0.05). The levels decreased on day 14, with a statistically significant difference between the 3% COS + Z-C group and the blank group (*p* < 0.05), demonstrating that fibroblast growth factor was more abundant during the cell proliferation phase and less abundant during the tissue remodeling phase. The higher content of fibroblast cytokines on day 7 and the greater change in content after 14 days proved that these metal ion cross-linked gel films could indeed promote cell proliferation [40].

New blood vessels can transport various nutrients and cytokines needed for the tissue healing process [41]. CD31 is a marker of neointima formation, and can be used to assess neointima formation [42]. The analysis showed that the expression levels of CD31 and FGF were higher in each group on day 14 than on day 7 (Figure 7B). The 3% COS + Z-C group was significantly different from the blank and positive control groups at day 7 (*p* < 0.05). Interestingly enough, the AOD value was as high as 25.25% on day 14, showing that the 3% COS + Z-C group had a good function of promoting trabecular neovascularization. This may be due to the synergistic effect of the addition of multiple ions, which together activate angiogenic factors and promote the formation of new blood vessels [43]. It was further demonstrated that metal ion-containing binary-network gel films, especially bimetallic ion cross-linked gel films, can effectively promote wound healing.

## 3. Materials and Methods

### 3.1. Experimental Materials

Sodium alginate (SA, M_w_ = 460,000 g mol^−1^), chitooligosaccharide (COS, molecular weight < 2000 Da), SrCl_2_, CaCl_2_ and ZnCl_2_ were purchased from Shanghai Maclean Biochemical Co. The Yunnan Baiyao (YN) was purchased from Yunnan Baiyao Group Co. The acridine orange (AO)/ethidium bromide (EB) staining kit was purchased from Biotechnology (Shanghai, China) Co. The H&E staining kits were purchased from Solaibao (Beijing, China). *Escherichia coli* (*E. coli*, CCTCCAB93154) and *Staphylococcus aureus* (*S. aureus*, ATCC9118) were purchased from China Type Culture Collection Center. The HaCaT cells were purchased from Suzhou Benetton Biotechnology Co Ltd. (Suzhou, China). SPF weight 20 ± 2 g male mice were purchased from Hebei Fu (Beijing, China) Biotechnology Co Ltd. (Beijing, China) 20190010. All of the chemicals were analytical degree reagents, and were used as received.

### 3.2. Preparation of Ion^2+^-COS/SA Gel Films 

COS with a molecular weight < 2000 Da was accurately weighed and diluted in water in order to prepare aqueous chitosan solutions respectively with concentrations of 1%, 3% and 5%. The pH of the solution was adjusted to 5.6 and stirred in a water bath at 40 °C for 1 h. Then, 1% CaCl_2_ solution was added to each of the three concentrations of COS, and 1% ZnCl_2_ and 1% SrCl_2_ solutions were added to 3% COS and stirred at room temperature for 1 h. The coordination products of COS and the metal ions were prepared for further use. In total, 10 mL SA at a concentration of 2% (mass:volume) was poured into prefabricated molds (10 cm × 5 cm × 1 cm Teflon plates). In total, 5 mL COS–metal ion coordination product was filled into a medical spray bottle (20 mL), and the thickness of the composite gel films was controlled by the number of sprays. In order to cross-link as fully as possible, the number of sprays of the metal ion solution was determined to be 15 times higher. Interestingly, the gel films take only 5 s to form a film. After the film formation, the spraying of Ca^2+^ solution was continued on the film spiked with Zn^2+^ and Ca^2+^; we then waited for film formation.

The resulting gel films were named 1% COS + Ca^2+^ (renamed as 1% COS + C), 3% COS + Ca^2+^ (renamed as 3% COS + C), 5% COS + Ca^2+^ (renamed as 5% COS + C), 3% COS + Zn^2+^−Ca^2+^ (renamed as 3% COS + Z-C), and 3% COS + Sr^2+^−Ca^2+^ (renamed as 3% COS + S-C).

### 3.3. SEM Morphological Analysis

The microstructure and morphology of the samples were measured using a NovaNanoSEM450 (FEI Technologies, Hillsboro, OR, USA) field emission scanning electron microscope (FESEM). Energy dispersive spectroscopy (EDS) was performed using a Quanta 250 FEG (FEI, Hillsboro, OR, USA).

### 3.4. FTIR

The infrared absorption spectra of the samples were measured in the range of 400–4000 cm^−1^ using an IRSpirit-1 infrared spectrometer (Shimadzu, Japan) at a resolution of 4 cm^−1^ and a scanning frequency of 32 times.

### 3.5. XRD

The prepared samples were measured by XRD (Ultima, Rigaku, Japan). The diffraction intensity data were scanned in the range of 2θ of 20°–80°, with a sampling step of 0.02°.

### 3.6. Contact Angle

In order to detect the hydrophilicity of the support surface, the static contact angle was measured using the static drop method and a video-based contact angle system (OCA20, Data Physics, Filderstadt, Germany).

### 3.7. Mechanical and Rheological Property Testing

The polymer films were cut into rectangular specimens of 20 × 10 mm, and each COS/SA/M^2+^ PDF was tested in tension using a mass spectrometer (TMS-PRO, FTC, Erie, PA, USA) at a stretching rate of 2 mm/min. The tests were carried out at 25 °C using a rheometer (HAAKE MARS III, Thermo, Waltham, MA, USA). The frequency scan experiments were performed in the range of 0.1–100 rad at 1% strain, and the corresponding values of the storage modulus (G’) and loss modulus (G”) were recorded.

### 3.8. Inflationary Behavior

After the weight of the dry film (*Wd*) was estimated, the wet films were prepared by soaking in distilled water for 1 h, 2 h, 3 h, 6 h, and 24 h. Next, the surface water was absorbed, and the weight of each membrane (*Ww*) was estimated. The swelling of each membrane was determined by the following equation:

$$Swelling\;degree\;(\%)=\frac{Ww-Wd}{Wd}\times 100\%$$

where *Ww* and *Wd* denote the weight of the wet film and dry film, respectively.

### 3.9. Porosity Measurements

The porosity of the films was determined by the liquid displacement method. The weight of the films (1 cm × 1 cm) was recorded, and then the films were placed in a beaker containing 5 mL anhydrous ethanol (99%) for 24 h until equilibrium was reached. The gel film was then taken out and weighed again. Its porosity was calculated as follows:

$$Porosity\;(\%)=\frac{W2-W1-W3}{W2-W3}\times 100\%$$

where *W1* is the initial weight of the film, *W2* is the sum of the weight of the submerged film and the anhydrous ethanol, and *W3* is the weight of the anhydrous ethanol after the film is removed.

### 3.10. Thermal Stability

The thermal stability and dynamic properties were measured using a thermal analyzer TGA/DSC1/1600LF (Mettler Toledo, Zurich, Switzerland), heated from 35 °C to 800 °C at a heating rate of 10 °C/min with a constant nitrogen purge.

### 3.11. Antioxidant Activity Analysis

#### 3.11.1. FRAP Analysis

In total, 5 μL of the membrane samples and 180 μL FRAP reagent were mixed in a 96-well plate. The microplates were then incubated at 37 °C for 3–5 min, and the absorbance of the mixture was obtained at 593 nm using an enzyme marker (VersaMax, Molecular Devices, San Jose, CA, USA).

#### 3.11.2. DPPH Free Radical Scavenging Activity

The DPPH radical scavenging activity of the films was determined using a previously reported technique [44]. The reaction mixture was prepared by mixing 2 mL of samples of different concentrations with 2 mL DPPH solution (0.1 mM, in 95% methanol). Ascorbic acid was used as a positive control. The mixture was incubated in the dark at room temperature for 30, and then placed under an enzyme calibrator (VersaMax, Molecular Devices, San Jose, CA, USA.) and the absorbance at 517 nm was measured.

#### 3.11.3. Ability to Scavenge ABTS Free Radicals

The ABTS radical scavenging ability of the films was tested using the technique of Re et al. [45]. The ABTS^+^ solution was prepared by mixing ABTS solution (0.7 mM) with an equal amount of potassium persulfate (2.45 mM). The absorbance at 734 nm was 0.70 ± 0.05 using a phosphate buffer solution (5.0 mM, pH 7.4) to dilute the ABTS^+^ solution after 16 h of incubation at room temperature in the dark. As a result, 5.0 μL of the diluted ABTS^+^ aliquot was added to 0.2 mL of the sample, using the phosphate buffer solution as a control. After 10 min of incubation, the absorbance of the samples was measured at 734 nm (VersaMax, Molecular Devices, San Jose, CA, USA.).

### 3.12. Antibacterial Activity Analysis

The inhibition of the bacterial growth of *E. coli* and *S. aureus* in vitro by the blank gel film and composite film was evaluated using the disc diffusion method. Briefly, *E. coli* and *S. aureus* colonies were removed using an inoculation loop, pressed against the wall of a 3-mL saline bottle, and dissolved in saline. A swab soaked in the bacterial suspension was applied uniformly to the plate until a bacterial biofilm was formed. A blank gel film and a composite film of a uniform 2 cm in diameter were placed on the bacterial biofilm, and were placed in a shaker incubator rotating at 50 rpm at 37 °C for 24 h. Afterwards, the zone of inhibition was measured using Vernier calipers.

### 3.13. Cell Viability Assay

HaCaT cells were inoculated in 96-well plates at an inoculation density of 10,000 cells/well, and were cultured in gel film extract. The 96-well plates were incubated in an incubator at 37 °C with 5% CO_2_. Acridine orange (AO)/ethidium bromide (EB) mixed fluorescent staining solution was prepared by mixing equal amounts of equal concentrations of AO solution and EB solution. After the HaCaT cells were respectively cultured to 12 h, 48 h and 36 h, the HaCaT cells were dissociated by adding an appropriate amount of trypsin in the 96-well plate. We then aspirated 20 μL of HaCaT cell suspension and added an appropriate amount of AO/EB mixed fluorescent staining solution, and observed the morphology of the HaCaT cells on the slide using a fluorescent inverted microscope.

### 3.14. In Vivo Healing Promotion Experiment

#### 3.14.1. Construction of the Trauma Models

The animal experiments were approved by the Ethics Committee of Shandong University Weihai Municipal Hospital (2021034, approved on 2 May 2021). The gel film with the best overall performance was first selected based on the above performance characterization for the next step of the in vivo healing promotion experiments. Mice were randomly divided into 3 groups of 9 mice each, including a blank group, a positive control group, and a 3% COS + Z-C group. The blank group was left untreated, and the dressing used in the positive control group was Yunnan Baiyao. After 1 week of adaptive feeding, the anesthetized mice were injected intraperitoneally with 1% pentobarbital sodium at 50 mg/kg. The hair was shaved from the dorsal region to expose the dorsal skin, and the skin surface was disinfected with 70% alcohol. A full skin wound with a diameter of 0.8 cm was then formed.

#### 3.14.2. Histological Assessment

Regenerated skin tissue specimens were respectively collected on days 3, 7 and 14 after injury. The tissue sections were fixed with 4% paraformaldehyde and embedded with paraffin. The sections were then stained with H&E, followed by microscopic observation (Axio Observer, Zeiss, Baden-Württemberg, Germany).

#### 3.14.3. Immunohistochemistry Analysis

The expression of fibroblast growth factor-10 (FGF-10) and PECAM-1 (CD31) was detected by immunohistochemistry. Tissue sections were collected, dewaxed, and placed in distilled water in order to repair the antigens. Endogenous peroxidase was blocked by 3% H_2_O_2_-methanol solution, and the antigen was recovered with citrate buffer (pH 6.0). Sections were then sealed and incubated with 50–100 μL primary antibody (50–100 μL diluted primary antibody) for 2 h at 37 °C in a humid atmosphere. After being washed with PBS, the sections were incubated at 37 °C for 30 min, followed by the addition of a universal IgG Antibody-Fab fragment-HRP multimer (50 μL). Next, after washing with PBS, the sections were viewed under a light microscope (Axio Observer, Zeiss, Baden-Württemberg, Germany), after which the photographs were analyzed using Image J (Media Network Technologies, Inc., Rockville, MD, USA).

### 3.15. Statistical Analysis

The experimental data were expressed as the mean ± standard deviation (mean ± S.D). SPSS (version 20) was used for the statistical analysis. Multiple comparisons were performed between the groups using the LSD method, with *p* < 0.05 as the level of significance. Relevant data were calculated from the experimental images using Image J (National Institutes of Health, Bethesda, MD, USA).

## 4. Conclusions

We synthesized physical double-network gel films cross-linked with metal ions with the optimal COS content using an in situ spray method. The mechanical properties were improved by introducing divalent ions into the gel films. Among the single-ion cross-linked gel films, the calcium-containing gel film with 3% COS content had the best mechanical properties and strength, and significant antibacterial ability, cell viability, percentage of wound healing, granulation tissue and angiogenesis. Thus, the in situ prepared 3% COS + Z-C cross-linked gel film was a very suitable and promising fully bio-based wound dressing. Based on this, the addition of multiple metal ions to different gel films by casting and the in-situ spray method can be attempted in future studies, which could reduce or even avoid the toxic side effects of gel films, and could conveniently prepare gel films with mechanical properties and healing-promoting effects, bringing insight to the safe and efficient preparation of wound dressings.

## Data Availability

Not applicable.
